# Obituary.—Dr. Chas. A. Weston, Nicolaus, Cal.

**Published:** 1872-05

**Authors:** 


### Editorial.

Obituary.–Died at Nicolaus, SutterCo., Cal., Feb'ry 25th, Dr. Chas. Weston, late Surgeon U.S.A. Dr. Weston was born in Charleston, May 20th, 1840, and graduated in Jefferson Medical College, Philadelphia in 1861. He then commenced practice in that city, but shortly after was commissioned as Surgeon in the U. S. Service. He received a wound in the region of the heart, while in discharge of professional duties, from a shell thrown into the hospital. The Doctor came overland to California in search of health, and so far regained it as to be able to resume the practice of his profession, to which he has ardently attached, and in the active duties of which he continued to within three days of his death. When conscious of his approaching end, he arrranged his business for the event, and suddenly expired in the arms of a friend, of whom he had requested to be raised up in bed. He leaves a devoted wife to mourn his death, and the community while they sympathize with her, also feel their loss in an able and beloved physician.

